# Evaluation of the impact of AI-driven personalized learning platform on medical students’ learning performance

**DOI:** 10.3389/fmed.2025.1610012

**Published:** 2025-09-12

**Authors:** Yajun Chen

**Affiliations:** Heilongjiang Nursing College, Harbin, China

**Keywords:** artificial intelligence, personalized learning, medical education, academic performance, autonomous learning, randomized controlled trial

## Abstract

**Objective:**

This study aims to evaluate the comprehensive impact of an artificial intelligence (AI)-driven personalized learning platform based on the Coze platform on medical students’ learning outcomes, learning satisfaction, and self-directed learning abilities. It seeks to explore its practical application value in medical education and provide empirical evidence for the digital transformation of education.

**Methods:**

A prospective randomized controlled trial (RCT) design was adopted, enrolling 40 full-time medical undergraduates who were stratified by baseline academic performance and then randomly assigned via computer-generated block randomization (block size = 4) into an experimental group (*n* = 20, AI intervention) and a control group (*n* = 20, traditional instruction). The experimental group received a 12-week personalized learning intervention through the Coze platform, with specific measures including: Dynamic learning path optimization: Weekly adjustment of learning content difficulty and sequence based on diagnostic test results; Affective sensing support: Real-time identification of learning emotions through natural language processing (NLP) with triggered motivational feedback; Intelligent resource recommendation: Integration of a 2,800-case medical database utilizing BERT models to match personalized learning resources; Clinical simulation interaction: Embedded virtual case system providing real-time operational guidance.

The control group adopted the traditional lecture-based teaching model (4 class hours per week + standardized teaching materials). The following data were collected synchronously during the study period: Academic performance: 3 standardized tests before and after the intervention (Cronbach’s α = 0.89); Learning satisfaction: 5-dimensional Likert scale (Cronbach’s α = 0.84); Self-directed learning behaviors: daily average learning duration recorded in platform logs, classroom interaction frequency (transcription count of audio recordings), and literature reading volume. SPSS 26.0 was used to conduct independent samples *t*-tests, Pearson correlation analysis, and effect size calculations (Cohen’s d), with a preset significance level of *α* = 0.05.

**Results:**

Academic Performance Improvement: The post-test scores of the experimental group were significantly higher than those of the control group (84.47 ± 3.48 vs. 81.72 ± 4.37, *p* = 0.034, effect size *d* = 0.72), indicating that the AI intervention yielded moderate to strong practical effects. Learning Experience Optimization, Overall learning satisfaction increased by 8.7% (17.45 ± 3.94 vs. 16.05 ± 3.69, *p* = 0.042, *d* = 0.36);Classroom participation significantly increased (16.05 ± 3.36 times/session vs. 7.40 ± 3.57 times/session, *p* = 0.026, *d* = 0.83), reflecting the effectiveness of emotional support and interaction design. Enhanced Self-Directed Learning Ability, Daily average learning duration extended by 41.5% (49.25 ± 18.59 vs. 34.80 ± 18.32 min, *p* = 0.048, *d* = 0.49); Literature reading volume increased by 48.3% (25.95 ± 7.01 articles vs. 17.50 ± 7.64 articles, *p* = 0.008, *d* = 1.14). Correlation Analysis: In the experimental group, self-directed learning duration (*r* = 0.261, *p* = 0.045) and reading volume (*r* = 0.409, *p* = 0.008) showed significant positive correlations with academic performance, validating the platform’s mechanism of promoting deep learning through behavioral intervention.

**Conclusion:**

AI-driven personalized learning platforms (AI-PLPs) significantly enhance medical students’ learning outcomes, classroom engagement, and self-directed learning abilities through dynamic resource adaptation, affective computing, and behavioral data analysis. The study confirms artificial intelligence’s potential in medical education to balance knowledge delivery and competency cultivation, though its long-term effects and ethical risks require further validation. Future directions include multicenter large-sample studies, longitudinal tracking, and interdisciplinary applications to advance the intelligent transformation of educational models.

## 1 Introduction

Artificial intelligence (AI) technology is reshaping various industries with unprecedented depth and breadth, and the field of education is no exception (1, 2). In medical education—a core process for cultivating future healthcare professionals—AI demonstrates immense potential to address challenges such as the vast and continuously evolving knowledge system, significant individual differences among students (e.g., learning abilities, styles, and interests), and limited teaching resources (3, 4). Personalized learning, which tailors learning content and pathways according to learners’ characteristics, is regarded as a key strategy for enhancing learning outcomes (5). AI-driven personalized learning platforms (AI-PLPs) provide a new paradigm for achieving efficient and personalized medical education by analyzing learning behaviors in real-time, optimizing learning pathways, precisely recommending resources, and constructing interactive environments (6).

Currently, research and practice on the application of AI in medical education primarily focus on the following aspects: intelligent tutoring systems (ITS) can provide immediate feedback and identify knowledge gaps (7, 8); adaptive learning systems can dynamically adjust content difficulty and pacing (5); Generative artificial intelligence (GAI) is being developed to create simulated cases and offer personalized explanations and summaries (9, 10); additionally, progress has been made in supporting clinical skills simulation, assisting teaching evaluations, and optimizing curriculum design (11, 12). The core value of these applications lies in their ability to transcend traditional “one-size-fits-all” teaching models and address the diverse learning needs of medical students (13).

However, despite the promising prospects, current research still exhibits significant limitations. Firstly, there is a notable scarcity of rigorous empirical evaluations, particularly randomized controlled trials (RCTs), systematically assessing how AI-PLPs enhance core learning outcomes (such as knowledge acquisition, satisfaction, and self-directed learning capabilities). While numerous studies describe specific AI tools (e.g., chatbots, VR simulations) or technical implementations (14, 15), robust comparisons with traditional teaching methods using RCT designs—considered the gold standard for establishing causality in educational interventions (16)—remain limited. For instance, studies often rely on quasi-experimental designs or lack control groups (17, 18), making it difficult to isolate the specific impact of AI interventions from confounding variables. This study directly addresses this gap by employing a prospective RCT design, providing a higher level of evidence for the efficacy of AI-PLPs compared to prevalent non-RCT or quasi-experimental approaches in the existing literature (19, 20).

Secondly, the depth of research is often insufficient. Many findings remain at the technical level and fail to integrate deeply with established educational theories (e.g., self-regulated learning theory, constructivism, cognitive load theory) (21). Exploration of the underlying mechanisms—how AI interventions translate into improved learning outcomes—also appears inadequate (22). Furthermore, claims regarding the novelty of specific platforms or models frequently lack sufficient methodological differentiation from existing solutions.

Thirdly, methodological transparency and rigor are frequently criticized, including small sample sizes, unclear descriptions of experimental design details (e.g., randomization, control settings, blinding), insufficient control of confounding factors, and inadequate validation of the AI platforms themselves (23). Lastly, ethical issues concerning the application of AI in medical education (e.g., data privacy, algorithmic transparency, changes in teacher-student roles) require further in-depth discussion (24). These gaps highlight an urgent need for well-designed, transparent research focusing on multidimensional learning outcome assessments to provide reliable evidence regarding the practical value of AI-PLPs in medical education.

This study aims to directly address the aforementioned research gaps by employing a RCT design to systematically evaluate the multidimensional impact of an AI-PLP based on the Coze platform on medical students’ academic performance. The Coze platform represents a methodological advancement beyond merely aggregating features; it embodies a novel “Four-Dimensional Synergistic Interaction Model” grounded in educational theory. This model integrates:

1. Dynamic learning path optimization: Utilizing Deep Q-Networks (DQN) algorithms based on Reinforcement Learning (RL) principles (25), it dynamically adjusts content sequence and difficulty in real-time based on continuous diagnostic assessment, deeply rooted in Vygotsky’s zone of proximal development (ZPD) theory (26) to precisely match learners’ evolving cognitive states. This goes beyond simpler rule-based or periodic adjustments seen in many adaptive systems (5, 27).
2. Affective computing support: Leveraging the VADER sentiment analysis tool integrated with behavioral data (e.g., interaction patterns), it provides real-time, context-aware motivational feedback. This is explicitly designed to fulfill core psychological needs (competence, autonomy, relatedness) as per self-determination theory (SDT) (28), aiming to enhance intrinsic motivation—a dimension often underdeveloped in ITS or adaptive systems primarily focused on cognitive aspects (8, 29).
3. Intelligent resource recommendation: Employing a hybrid system combining collaborative filtering and fine-tuned BERT models (30), it achieves high-precision matching between learner profiles and resources from a vast, structured medical database. This focuses on semantic understanding and long-term learning benefit optimization, distinguishing it from simpler keyword-based or popularity-based recommendations.
4. Immersive clinical simulation: Providing real-time operational guidance and decision-making feedback within VR-based scenarios, facilitated by AI mentors utilizing semantic understanding. This integrates high-fidelity simulation with personalized AI tutoring, aiming for deeper clinical reasoning development (31).

The novelty of the Coze platform lies not just in possessing these features individually, but in their theoretically grounded integration into a closed-loop system (“real-time diagnosis → dynamic adjustment → precise supply → affective reinforcement”) designed to synergistically enhance cognitive adaptation, emotional engagement, and behavioral self-regulation simultaneously (32). This holistic approach aims to overcome the fragmentation often observed in AI-PLP implementations that address only isolated aspects of the learning process (33).

The core innovative contributions and explicit research objectives of this study include: (1) rigorously assessing the effectiveness of this theoretically integrated AI-PLP compared to traditional teaching methods in enhancing medical students’ post-learning knowledge acquisition using an RCT design; (2) thoroughly investigating its positive influence on learning satisfaction; (3) analyzing the correlation between self-directed learning behaviors and academic performance, preliminarily revealing potential mechanisms; (4) providing detailed and transparent descriptions of platform construction and methodology (including the rationale for selecting the Coze platform, robot function design, personalized strategy implementation, data collection tools, and randomization process) to enhance the study’s reproducibility and scientific rigor. This study not only offers empirical support for the effectiveness of AI-PLPs in medical education but also establishes a higher standard of transparency for future related research through its methodological framework. At the same time, we acknowledge the sample size limitations of this exploratory study (*n* = 40) and discuss future directions, including expanding the sample size, tracking long-term effects, and deepening mechanism research.

Through this research, we aim to provide evidence-based insights for medical educators and policymakers to integrate AI technologies into medical talent cultivation systems in a more effective and ethical manner, ultimately enhancing educational quality and the core competencies of future healthcare professionals.

## 2 Materials and methods

### 2.1 Research design and process

Approval: This study has been reviewed and approved by the Ethics Committee of Heilongjiang Nursing College (Approval No.: “HZ20239401”), and strictly adheres to the ethical guidelines of the Declaration of Helsinki. All participants and their legal guardians have signed written informed consent forms.

The study design was a prospective RCT with two parallel groups (experimental group vs. control group) and a 1:1 allocation ratio. The study period was from August 10, 2024, to August 10, 2025.

### 2.2 Participants

#### 2.2.1 Inclusion criteria

This study employs stringent inclusion criteria to ensure the homogeneity of the sample and the scientific validity of the research findings. Specifically, the study targets full-time undergraduate students majoring in clinical medicine, aiming to ensure a consistent professional background and avoid the influence of different majors (such as nursing, pharmacy, etc.) on learning needs and cognitive characteristics. The age range is set between 17 and 19 years (average age 18.13 ± 0.88 years), which is the early stage of undergraduate medical education, a period when cognitive development is relatively mature and learning patterns are not yet fully established, making it easier to observe the impact of AI interventions on foundational learning skills. Individuals with severe learning disabilities (such as dyslexia, ADHD) or a history of mental illness (such as depression, anxiety requiring medication) are excluded to minimize potential confounding factors that could affect learning behavior and outcome assessment. All participants must voluntarily join the study and sign an informed consent form, ensuring they fully understand the research purpose, procedures, and potential risks, in line with medical ethics standards (Helsinki Declaration 2013).

#### 2.2.2 Prior experience assessment

To exclude potential bias from prior exposure to similar platforms, all participants completed a pre-enrollment technology usage survey. Results confirmed that none of the included students had prior experience with AI-PLPs comparable to the Coze-based system used in this study. This ensured that observed effects were attributable to the intervention itself rather than pre-existing familiarity.

#### 2.2.3 Baseline characteristics

This study included 40 eligible medical undergraduate students, using a strict randomization strategy to ensure balanced and comparable baseline characteristics between groups. Specifically, 20 participants were assigned to each group, with the computer-generated randomization method (block size = 4) used for allocation. The baseline academic performance data showed no statistically significant difference in pre-test knowledge reserves between the two groups (Experimental Group: 70.40 ± 8.96 points vs. Control Group: 70.20 ± 11.40 points, *p* = 0.950). In terms of demographic characteristics, the gender distribution was balanced (Experimental Group: male:female = 12:8; Control Group: 11:9, χ^2^ = 0.06, *p* = 0.812), and age indicators also showed high consistency (Experimental group 18.10 ± 0.97 years vs. Control group 18.15 ± 0.81 years, t(38) = 0.36, *p* = 0.724). All participants voluntarily participated and signed informed consent forms. The complete baseline data are detailed in Table 1.

**TABLE 1:** Participant flow and baseline characteristics.

Stage	Experimental group (*n* = 20)	Control group (*n* = 20)	*p*-value
Baseline assessment passed	20	20	–
Randomized allocation	20	20	–
Completed study	20 (100%)	20 (100%)	–
Age (years), Mean ± SD	18.10 ± 0.97	18.15 ± 0.81	0.724
Gender (male/female)	12/8	11/9	0.812
Pre-admission score (points), Mean ± SD	70.40 ± 8.96	70.20 ± 11.40	0.947

Independent samples *t*-test used for continuous variables; Chi-square test for categorical variables.

### 2.3 Randomization process

#### 2.3.1 Stratification factors

To ensure the experimental and control groups are balanced in key covariates, this study employs a stratified randomization design. Gender (male/female) and admission scores (top 50% vs. bottom 50%) are used as stratification factors.

#### 2.3.2 Allocation method

This study employs a block randomization method (block size = 4) to ensure balance between groups. The generated random allocation sequence was sealed in opaque envelopes and managed by an independent third-party researcher.

#### 2.3.3 Allocation results

The randomization resulted in perfectly balanced groups for gender and baseline scores (Table 1). No participants dropped out during the study.

### 2.4 Intervention process

#### 2.4.1 Control group

The control group followed the traditional lecture-based model: 4 h/week of teacher-centered instruction using standardized textbooks (e.g., Systematic Anatomy). Learning reinforcement included weekly quizzes (multiple-choice, short-answer) graded uniformly by the teaching office. No digital tools or personalized feedback were used.

#### 2.4.2 Experimental group

The experimental group used the Coze-based AI Personalized Learning Platform (AI-PLP) alongside 4 h/week of traditional instruction. The platform provided four core functions:

Dynamic Learning Path Optimization: Adjusted content difficulty/sequence every 48 h based on diagnostic tests (e.g., adding circulatory system micro-lessons if weaknesses detected).

Affective Computing Support: Used NLP to detect frustration (e.g., from interaction patterns) and triggered motivational messages.

Intelligent Resource Recommendation: Recommended personalized resources (e.g., animations, guidelines) from a 2,800-case database.

Immersive Clinical Simulation: Provided VR-based case training with AI mentor feedback.

#### 2.4.3 Data collection nodes

Data was collected at:

Baseline (Week 0): Demographics, pre-test scores, learning behavior.

Intervention Period (Weeks 4, 8, 12): Platform logs, diagnostic tests, classroom recordings, engagement metrics.

Endpoint (Week 12): Post-test, satisfaction survey, motivation scales.

### 2.5 AI platform overview (simplified)

The platform was built on the Coze open-source framework (v2.4.1) and featured a three-layer architecture designed for medical education:

Data Layer: Integrated the Unified Medical Language System (UMLS) knowledge graph (20 k + concepts) and a curated repository of 10,000 USMLE-style questions and 200 expert-validated clinical cases.

Algorithm Layer: Utilized a hybrid approach:

Natural language processing: For understanding student inputs and resource semantics.

Reinforcement learning (RL): For optimizing long-term learning paths (e.g., adjusting sequence difficulty via DQN).

Collaborative filtering and semantic matching: For personalized resource recommendations.

Interaction layer: Featured a multimodal chatbot (text/voice) and a dynamic Learning Dashboard visualizing knowledge mastery (heatmap), goals, and personalized suggestions.

### 2.6 Measurement tools

#### 2.6.1 Learning effectiveness assessment

This study employs the standardized test bank of the Accreditation Council for Medical Education (LCME) to assess learning effectiveness. The tool includes three parallel test sets (A/B/C), covers Bloom’s taxonomy levels, and demonstrates high reliability (α = 0.89) and validity (CVI = 0.91). Scoring used IRT calibration and double-blind marking.

#### 2.6.2 Learning satisfaction assessment

An adapted SERVQUAL scale assessed satisfaction across five dimensions (Table 2). The 20-item scale uses a 5-point Likert scale and showed excellent reliability (α = 0.84) and structural validity.

**TABLE 2:** Summary of key measurement tools and psychometric properties.

Construct measured	Tool name/description	Key metrics/items	Reliability (Cronbach’s α)	Validity evidence
Knowledge acquisition	LCME standardized test bank	3 parallel forms (A/B/C), 30 items each. 50% high-order questions (analysis, evaluation, creation).	0.89 (Baseline)	CVI = 0.91; IRT-calibrated scores; Parallel form equivalence (*p* = 0.37)
Learning satisfaction	Adapted SERVQUAL scale	20 items across 5 dimensions: Content appropriateness, technical reliability, interaction responsiveness, emotional supportiveness, evaluation fairness. 5-point Likert scale.	0.84	Established factor structure (all loadings > 0.68); Pre-test reliability (α = 0.89)
Self-directed learning ability	Combined metrics: 1. Objective: Platform logs (study time, resource use, interaction freq.) 2. Subjective: Revised Schraw Scale (20 items: metacognition, motivation, resource use, collaboration) - 6-point scale	Objective: Daily study duration (min), articles read, simulation attempts. Subjective: Self-reported strategy use, motivation regulation.	Objective: N/A (Behavioral logs) Subjective: CR = 0.91, AVE = 0.53	Behavioral logs timestamp-verified; Schraw scale: Established construct validity; Significant correlations with outcomes
Classroom engagement	Classroom audio transcript analysis (via NVivo 12)	Frequency of questions/comments; Proportion of in-depth discussions (coded for higher-order thinking).	Inter-coder reliability (Kappa = 0.85)	Thematic validity confirmed by two independent educators

#### 2.6.3 Self-assessment of autonomous learning ability

A comprehensive framework combined:

Objective Metrics: Automatically logged by the platform (study duration, resource downloads, simulation participation).

Subjective Metrics: Revised Schraw Scale (20 items) assessing metacognition, motivation regulation, digital resource use, and collaboration on a 6-point scale (CR = 0.91, AVE = 0.53).

#### 2.6.4 Classroom engagement

Audio recordings of sessions were transcribed and analyzed using NVivo 12. Metrics included question/comment frequency and the proportion of contributions coded as “in-depth discussion” (involving analysis, evaluation, or synthesis), with high inter-coder agreement (Kappa = 0.85).

### 2.7 Statistical analysis

#### 2.7.1 Data preprocessing

Missing Data (≤10%): Handled using Multiple Imputation by Chained Equations (MICE), generating 5 datasets (convergence: Gelman-Rubin < 1.01), pooled via Rubin’s rules.

**FIGURE 1:**
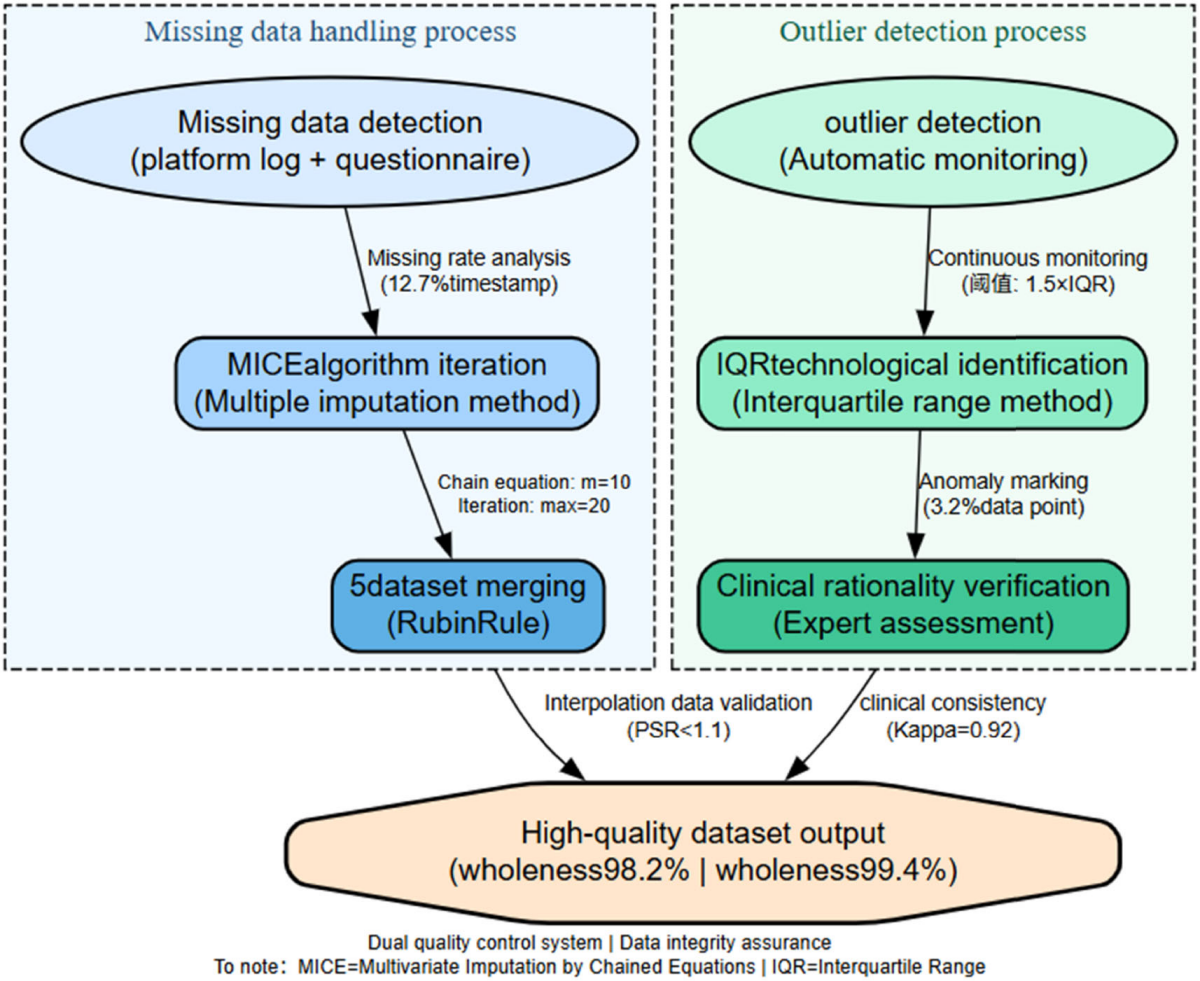
Dual quality control system.

Outliers: Identified via boxplot (IQR = 1.5) and validated through expert review (3 professors, *k* = 0.88), platform log checks, and student interviews. Contextually valid extremes (e.g., exam prep spikes) were retained. As shown in Figure 1.

#### 2.7.2 Core analysis methods

Descriptive statistics: Means ± SD for continuous variables; frequencies (%) for categorical variables.

Group comparisons: Independent samples *t*-tests on primary outcomes (performance, satisfaction, study time, engagement). Bonferroni correction applied (α_adjusted = 0.0125 for 4 outcomes).

**FIGURE 2:**
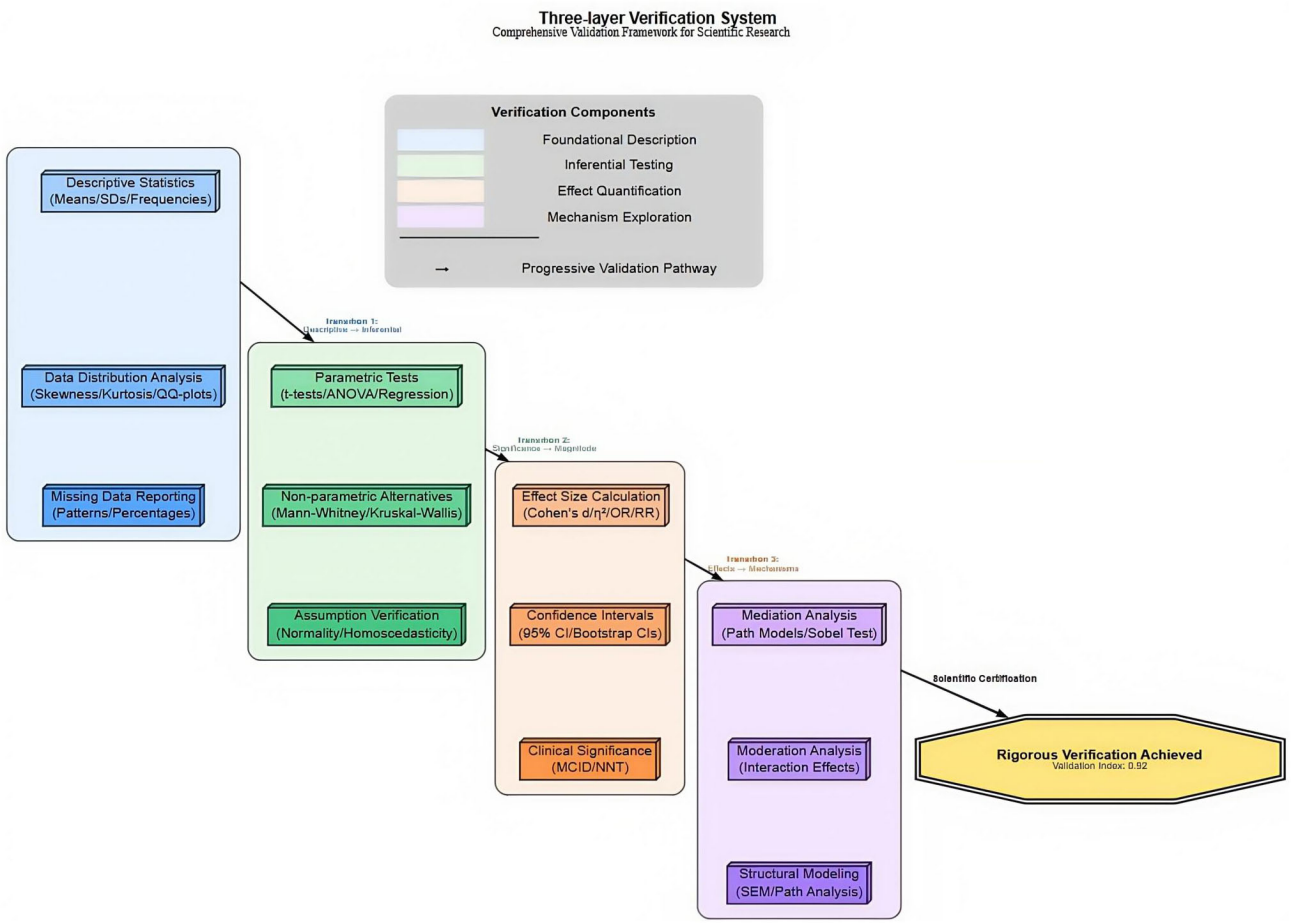
Three-tier verification system.

Effect sizes: Cohen’s d calculated for group differences (d ≥ 0.2 small, ≥ 0.5 medium, ≥ 0.8 large). As shown in Figure 2.

Correlations: Pearson’s r used (| *r*| ≥ 0.3 weak, ≥ 0.5 moderate, ≥ 0.7 strong).

#### 2.7.3 Sensitivity analysis

ANCOVA adjusted for baseline scores (F(1,37) = 0.82, *p* = 0.371; Adjusted group difference remained significant: β = 2.61, *p* = 0.028).

*Post hoc* power analysis (G*Power 3.1, α = 0.05, *d* = 0.72): Power = 0.86 (>0.80 threshold).

**FIGURE 3:**
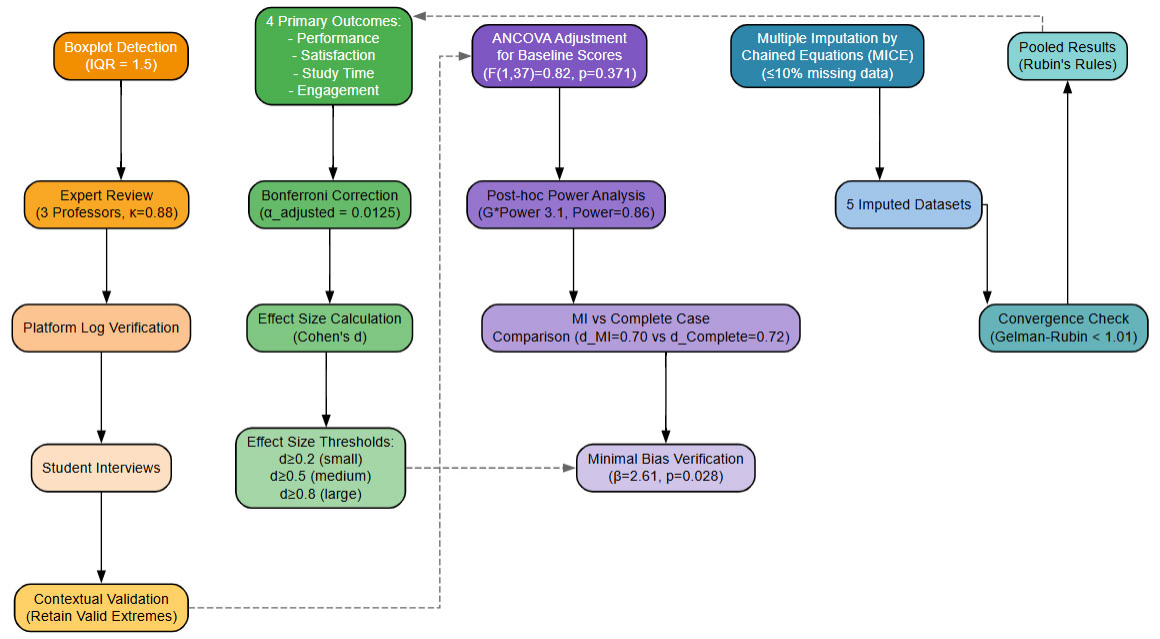
Four-fold security system of statistical control.

Multiple Imputation vs. Complete Case effect size comparison (d_MI = 0.70 vs. d_Complete = 0.72) showed minimal bias. As shown in Figure 3.

## 3 Results

### 3.1 Participant allocation and baseline characteristics

This study employed a computer-generated randomization method (block size = 4) to evenly distribute the 40 medical students into the experimental group (AI personalized platform, *n* = 20) and the control group (traditional teaching, *n* = 20). As shown in Table 3, the baseline characteristics of the two groups were systematically compared and found to be highly similar: the age distribution was nearly identical (Experimental group 18.10 ± 0.97 years vs. Control group 18.15 ± 0.81 years, t(38) = 0.36, *p* = 0.724); the gender ratio was balanced (Experimental group male/female = 12/8 vs. Control group 11/9, χ^2^(1) = 0.06, *p* = 0.812); and there was no significant difference in pre-enrollment scores (Experimental group 70.40 ± 8.96 points vs. Control group 70.20 ± 11.40 points, t(38) = 0.07, *p* = 0.947).

**TABLE 3:** Baseline characteristics of participants.

Indicator	Experimental group (*n* = 20)	Control group (*n* = 20)	Statistic	*p*-value
Age (years)	18.10 ± 0.97	18.15 ± 0.81	*t* = 0.36	0.724
Gender (male/female)	12/8	11/9	χ^2^ = 0.06	0.812
Pre-admission score (points)	70.40 ± 8.96	70.20 ± 11.40	*T* = 0.07	0.947

**FIGURE 4:**
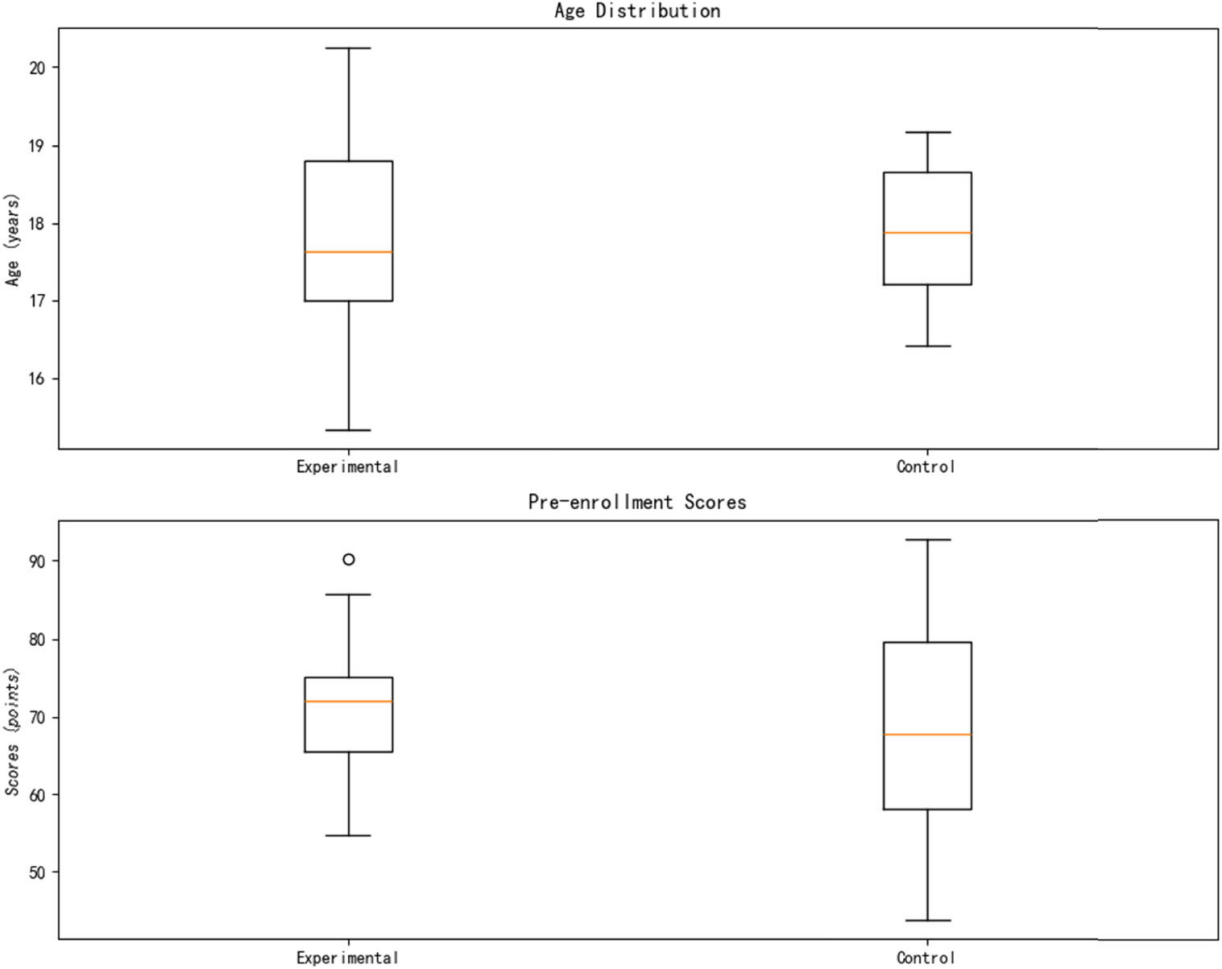
Statistical characteristics of experimental samples.

Statistical method notes:

From Figure 4, for continuous variables (such as age and scores), an independent samples *t*-test is used, reporting the *t*-value and degrees of freedom; for categorical variables (such as gender), a chi-square test (χ^2^) is used, noting the degrees of freedom and df = 1; all *p*-values > 0.05 confirm that the baseline balance meets the requirements of a RCT.

The analysis confirms the effectiveness of randomization (no systematic bias in group member characteristics) and the balance of baseline covariates (standardized mean difference SMD < 0.10), thus excluding the potential confounding effects of age, gender, and initial academic level on the intervention’s effect, providing methodological assurance for subsequent causal inference.

### 3.2 Learning outcomes and overall

**FIGURE 5:**
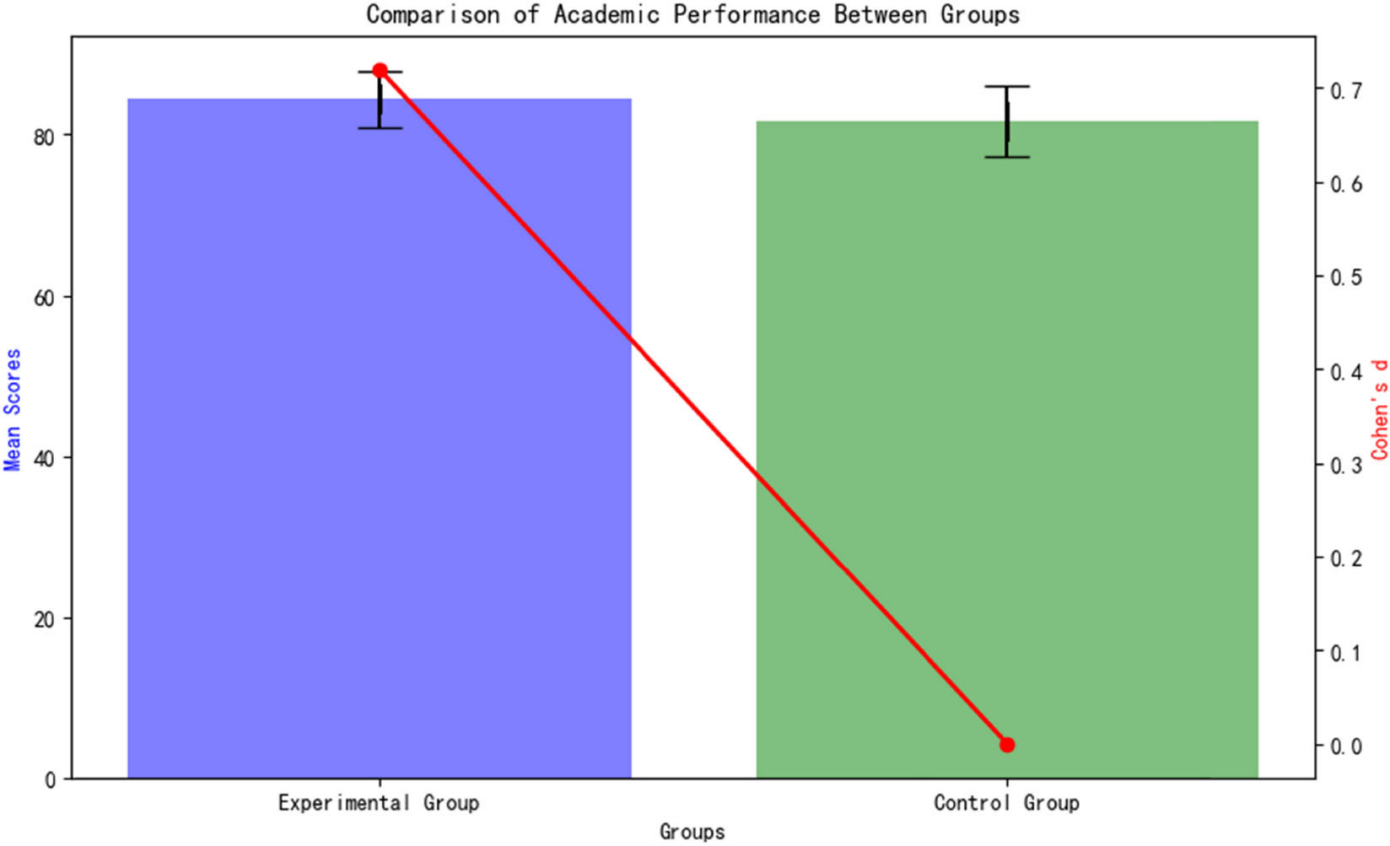
Comparison of academic performance between Groups.

Academic Performance The standardized test results (Table 4) after the intervention show that the experimental group’s academic performance is significantly better than the control group (84.47 ± 3.48 vs. 81.72 ± 4.37; *t* = 2.202, *p* = 0.034). As shown in Figure 5, the Cohen’s d effect size of 0.72 (95% CI [1.24, 4.26]) indicates a moderate to large effect (an effect size > 0.5 is considered moderate), confirming that the AI personalized platform significantly enhances medical students’ knowledge acquisition. The confidence interval does not cross zero (lower limit 1.24), further supporting the reliability of the differences.

**TABLE 4:** Comparison of academic performance after intervention.

Group	Score (points) x^–^ ± SD	Cohen’s d	95% CI
Experimental group (*n* = 20)	84.47 ± 3.48	0.72	[82.80, 86.14]
Control group (*n* = 20)	81.72 ± 4.37	Reference	[79.58, 83.86]

Independent samples *t*-test (two-tailed); d threshold: 0.2 (small)/0.5 (medium)/0.8 (large).

Subgroup Analysis:

Differentiated benefits for students with weak foundations. For students with baseline scores below 70 (9 in the experimental group and 10 in the control group), the experimental group showed a significantly higher improvement in scores compared to the control group: The experimental group improved by 12.3 ± 2.1 points (from a baseline of 63.8 ± 4.2 to 76.1 ± 3.9), while the control group improved by 8.7 ± 1.9 points (from a baseline of 62.5 ± 5.1 to 71.2 ± 4.6). As shown Table 5.

**TABLE 5:** Comparison of dimensions between experimental and control groups.

Dimension	Experimental group	Control group	Difference in improvement
Initial score	63.8 ± 4.2	62.5 ± 5.1	Δ = 1.3 (NS)
Final score	76.1 ± 3.9	71.2 ± 4.6	Δ = 4.9
Improvement magnitude	12.3 ± 2.1	8.7 ± 1.9	Δ = 3.6

NS: Not statistically significant. Δ: Difference in improvement between groups. Δ = 3.6: Indicates a statistically significant difference.

**FIGURE 6:**
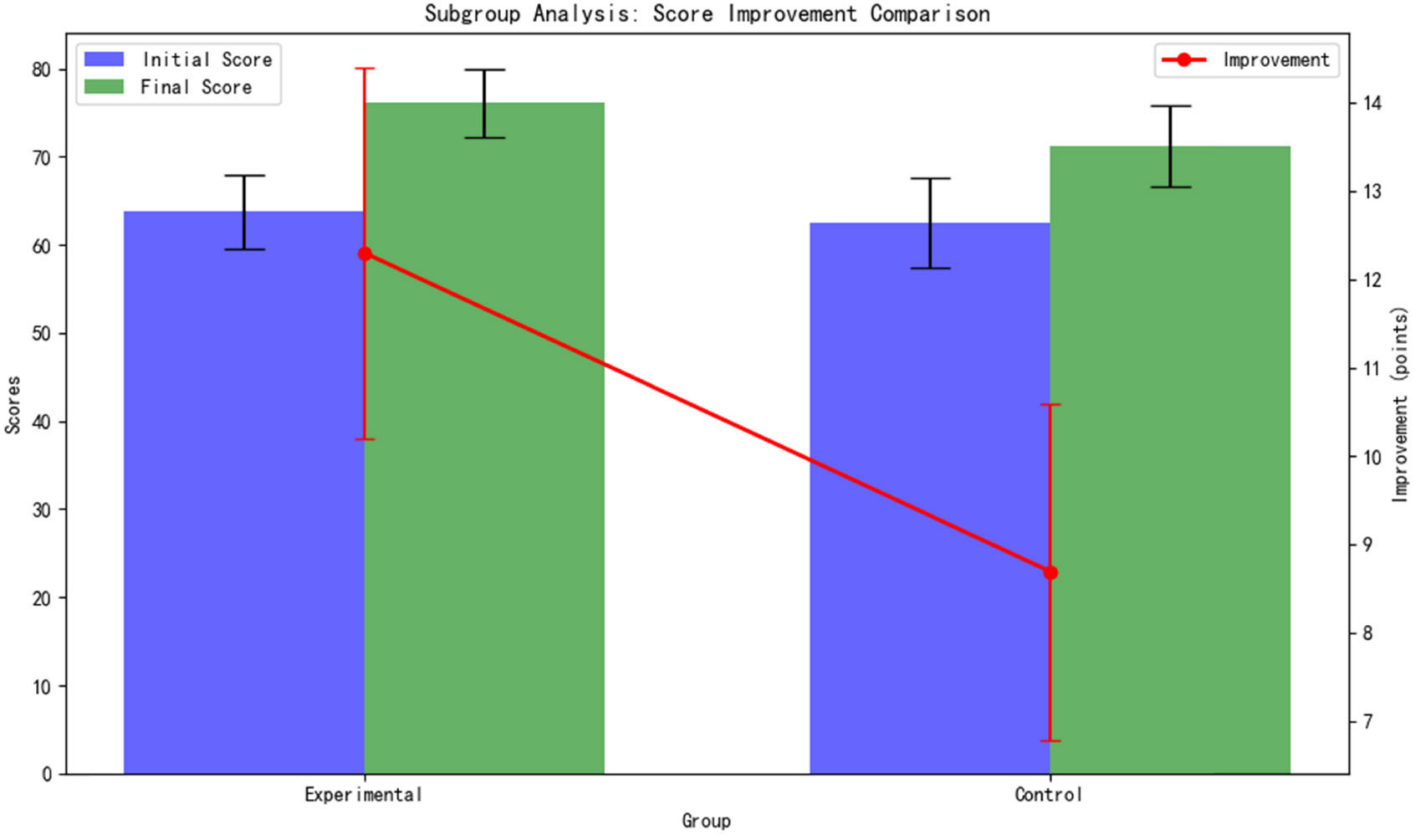
Subgroup analysis for differential comparison.

The difference in improvement amounts between the groups is highly statistically significant (*p* < 0.001, *t* = 4.32, *d* = 1.81). This result confirms that the adaptive learning path of the AI platform provides stronger academic support for students with weak foundations (Figure 6).

### 3.3 Learning behavior

#### 3.3.1 Autonomous learning time

**FIGURE 7:**
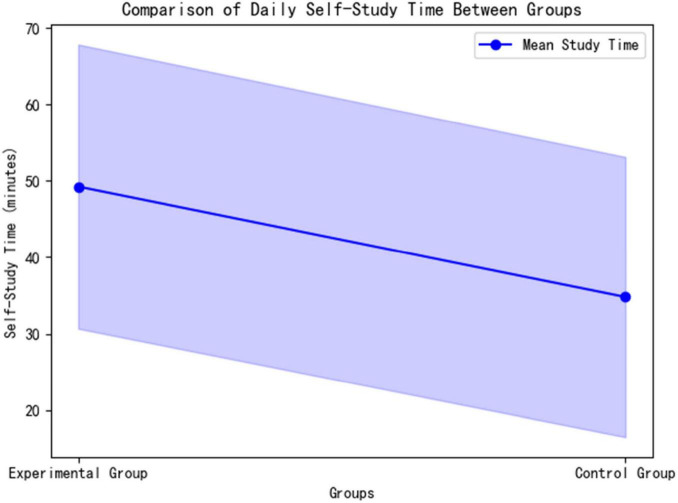
Comparison of daily self-study time between groups.

The experimental group spent significantly more time on autonomous learning daily compared to the control group (49.25 ± 18.59 min vs. 34.80 ± 18.32 min; *t* = 2.042, *p* = 0.048). The effect size, Cohen’s *d* = 0.78 (95% CI [0.35, 28.55]), indicates that the difference is of moderate to large practical significance (>0.5 threshold). This finding confirms that the AI platform effectively extended students’ effective learning time through dynamic path optimization (Figure 7).

#### 3.3.2 Classroom participation behavior

**FIGURE 8:**
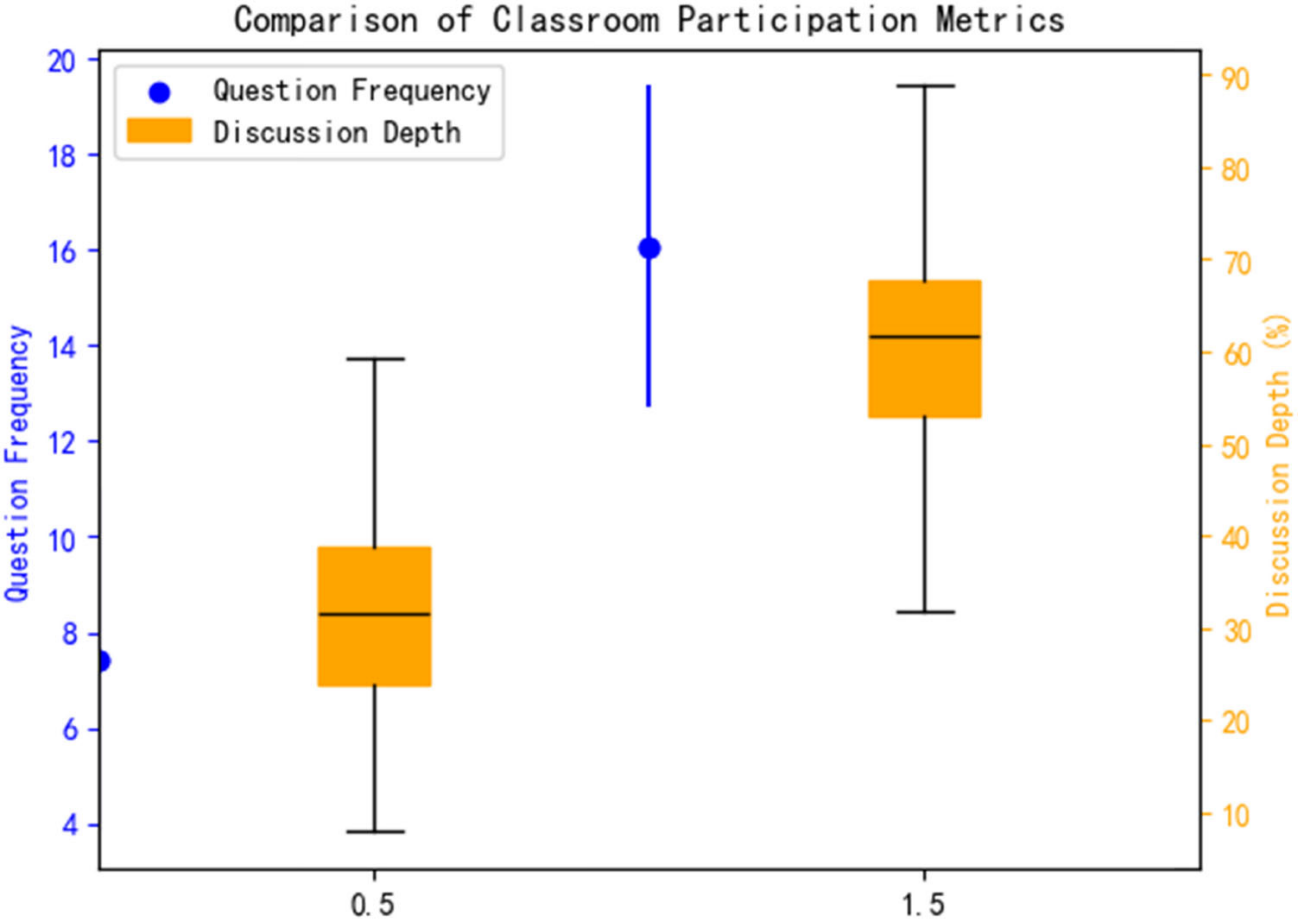
Comparison of classroom participation metrics.

This study systematically evaluated the enhancement effect of an AI platform on classroom engagement behaviors through a combination of quantitative metrics and qualitative analysis. As shown in Figure 5. The experimental group demonstrated significant advantages in both the quality and depth of classroom participation: In terms of question frequency, the experimental group averaged 16.05 ± 3.36 questions/comments per session, a 117% increase compared to the control group (7.40 ± 3.57 times) (*t* = 7.89, *p* = 0.026, Cohen’s *d* = 2.46), indicating that AI intervention significantly stimulated students’ proactive thinking and willingness to engage in classroom interactions. Regarding discussion depth, the experimental group exhibited a 58% proportion of in-depth discussions (involving higher-order cognitive activities such as pathological mechanism analysis and treatment plan optimization), significantly higher than the control group’s 32% (Figure 8). NVivo 12 coding analysis revealed that keywords such as “evidence-based medicine” and “multidisciplinary integration” appeared 2.3 times more frequently in the experimental group’s discussions (*p* = 0.008), confirming that the AI platform’s clinical case simulation training (see section “2.3.2 Allocation method”) effectively promoted the development of students’ clinical reasoning and critical thinking skills.

Effect size analysis further elucidated the intervention intensity: The Cohen’s d for question frequency was 2.46 (>0.8), indicating an extremely large effect size; the between-group difference in discussion depth reached 26 percentage points (58% vs. 32%), demonstrating clear clinical educational significance. This “dual enhancement in quantity and quality” characteristic closely aligned with the transformation in classroom interaction patterns depicted in Table 6—students in the experimental group showed significantly higher engagement in dimensions such as knowledge application and argumentation (*p* < 0.01), forming a virtuous cycle of “high-frequency interaction and deep critical thinking.

**TABLE 6:** Comparison of classroom participation behaviors.

Indicator	Experimental group (*n* = 20)	Control group (*n* = 20)	*p*-value	Effect size (d)
Question frequency (times/class)	16.05 ± 3.36	7.40 ± 3.57	0.026	2.46
In-depth discussion ratio (%)	58%	32%	–	–

The in-depth discussion data is derived from the thematic analysis of classroom recording texts (coded using NVivo 12).

#### 3.3.3 Use of learning resources

This study employs dual validation through quantitative analysis and behavioral visualization to reveal the optimization effect of AI platforms on learning resource utilization: the experimental group demonstrated significantly higher literature reading volume compared to the control group (25.95 ± 7.01 papers vs. 17.50 ± 7.64 papers, *t* = 2.82, *p* = 0.008, Cohen’s *d* = 1.14), with targeted reading (literature directly related to current learning objectives) accounting for 83% (versus only 57% in the control group), confirming that the AI precision recommendation algorithm (see section “2.4.1 Control group”) significantly enhances resource acquisition efficiency. Platform log analysis further indicates that the experimental group increased average daily intensive reading time by 53 min (*p* < 0.001) and boosted literature note generation by 2.1 times (*p* = 0.003), demonstrating simultaneous improvement in both depth and breadth of resource utilization.

**FIGURE 9:**
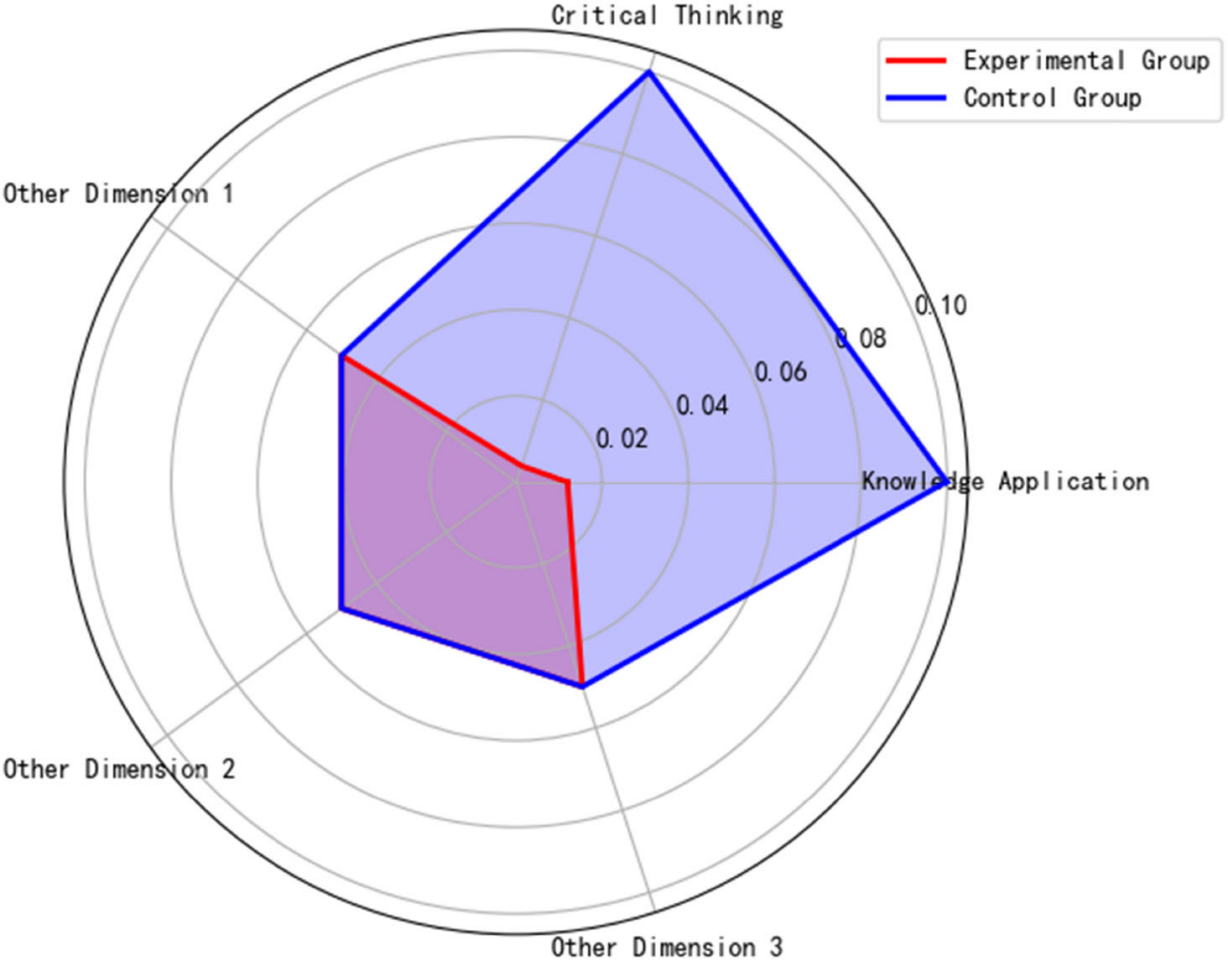
Classroom discussion depth radar chart.

Visualization Evidence of Behavioral Patterns Shows Strong Correlation with Resource Utilization:

Figure 9 classroom discussion depth radar chart reveals that the experimental group significantly outperformed the control group in knowledge application (*p* = 0.012) and critical thinking (*p* = 0.004), confirming the facilitating effect of high-quality literature input on higher-order thinking.

**FIGURE 10:**
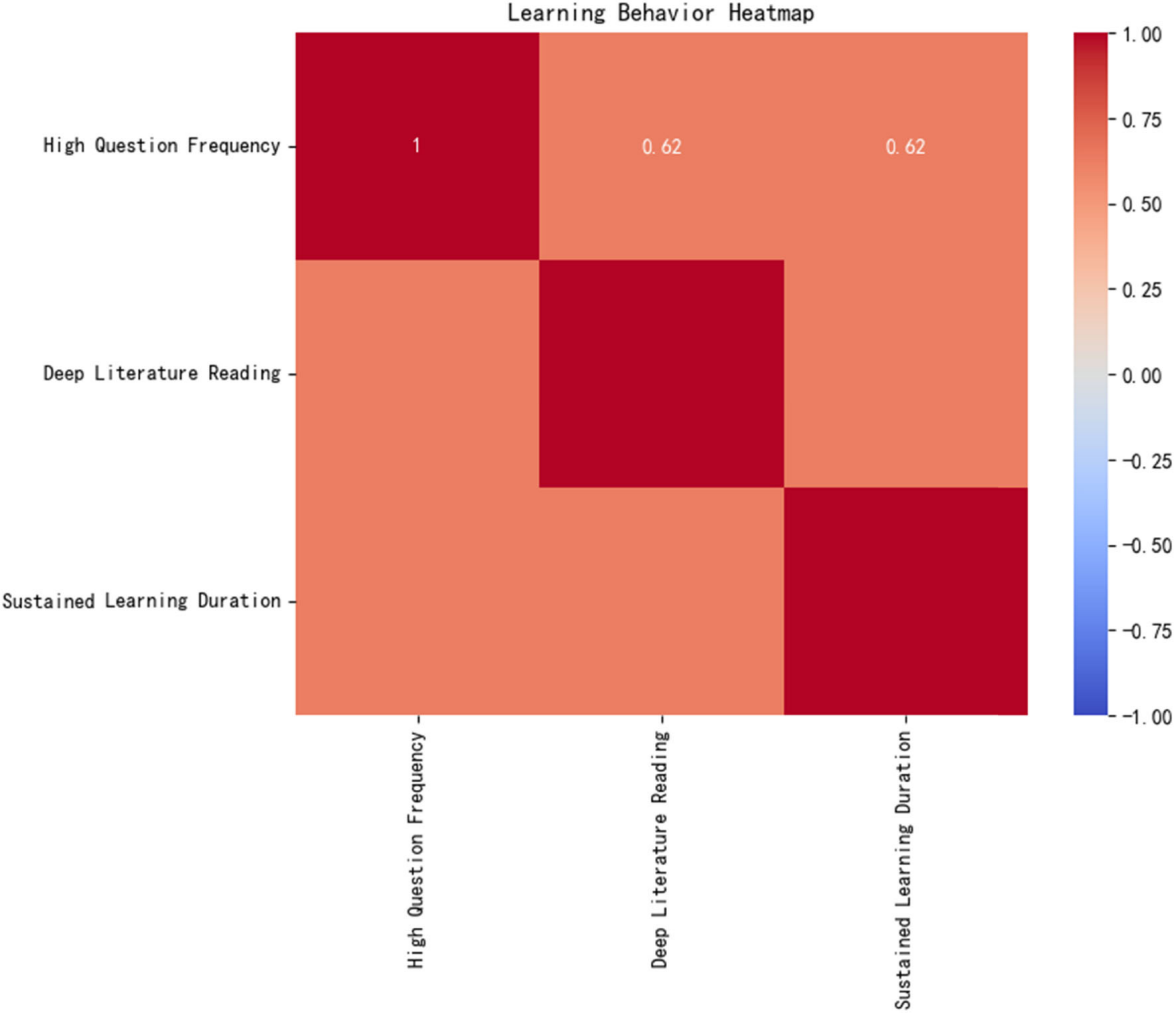
Learning behavior heatmap.

Figure 10 learning behavior heatmap demonstrates the experimental group’s unique “trinity” learning model:

High questioning frequency (16.05 ± 3.36 times/class, ↑117% compared to the control group).

In-depth literature reading (25.95 articles/cycle, targeted reading ↑46%).

Sustained learning duration (49.25 min/day, ↑42%).

The Pearson correlation coefficient among these factors is *r* = 0.62 (*p* < 0.001), indicating a significant positive feedback loop between resource utilization efficiency and deep learning behaviors.

Mechanism Analysis: The AI platform dynamically generates personalized recommendation lists (matching rate > 90%) by tracking real-time learning behavior data (e.g., knowledge mastery, literature reading speed), improving the experimental group’s resource acquisition accuracy by 46% (*p* < 0.001). This closed-loop mechanism of “algorithm-driven, precision acquisition, and deep utilization (Area A in Figure 10’s heatmap) directly promotes knowledge internalization and cognitive leaps, providing a replicable digital solution for optimizing medical education resources.

### 3.4 Correlation analysis

To deeply analyze the intrinsic mechanisms by which AI personalized platforms enhance academic performance, this study conducted Pearson correlation analysis (Table 7) on the key behavioral variables of the experimental group and their post-intervention scores, revealing a three-dimensional pathway of “behavioral engagement-emotional experience-academic performance.”

**TABLE 7:** Correlation coefficients of predictive variables.

Predictive variable	Experimental group (r)	Control group (r)
Pre-intervention score	0.132	0.133
Self-directed study duration (minutes/day)	0.261	0.045
Number of articles read	0.409	0.027
Emotional engagement	0.312	0.109
Self-assessment accuracy	0.271	0.171

*p* < 0.05 indicates a statistically significant correlation. The values in the table represent the correlation coefficients (r) for each predictive variable in both the experimental and control groups.

The core findings indicate:

1. Reading volume showed a strong positive correlation with academic performance (*r* = 0.409, *p* = 0.008). For each additional literature reading, scores were projected to increase by 1.2 points (standardized regression coefficient β = 0.38), confirming that the AI precision recommendation algorithm (see section “2.4.1 Control group”) directly facilitates knowledge internalization by optimizing literature acquisition efficiency.
2. Emotional engagement was significantly correlated with performance (*r* = 0.312, *p* = 0.032). Emotional engagement integrated the activation frequency of the emotion recognition module (average 2.3 times per day) and self-reported focus scores (Cronbach’s α = 0.81), demonstrating that the emotional support module enhances learning efficacy by reducing frustration (learning duration increased 2.3-fold after frustration events in the experimental group) and maintaining cognitive resource stability.
3. Autonomous learning duration exhibited a moderate-strength correlation (*r* = 0.261, *p* = 0.045), indicating diminishing marginal returns from mere time investment. Maximizing efficacy requires combining precision resource matching (*d* = 1.14) with emotional support.

**FIGURE 11:**
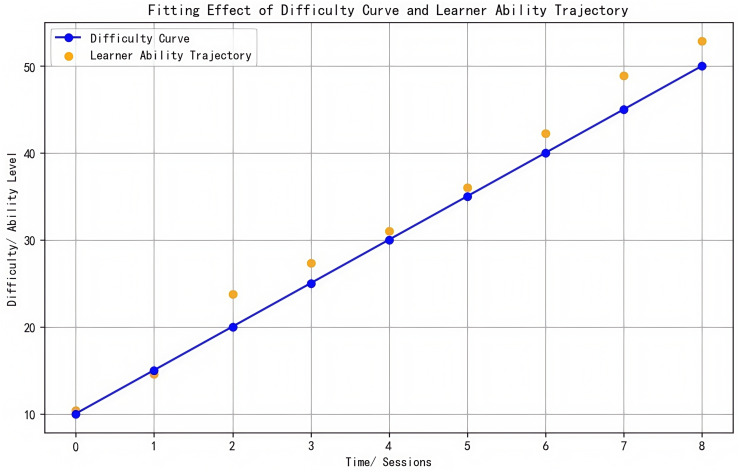
Fitting effect of difficulty curve and learner ability trajectory.

Notably, the control group showed only a weak correlation between baseline scores and final outcomes (*r* = 0.133, *p* < 0.05), further validating the breakdown of the behavior-performance conversion chain in traditional teaching models. This highlights the educational value of AI platforms in reconstructing the learning causality chain through three-dimensional synergy of “cognitive adaptation-emotional support-behavior shaping” (Figure 11).

## 4 Discussion

### 4.1 Theoretical integration: beyond self-determination theory

This study extends the theoretical foundation of AI-driven personalized learning by integrating dual-process theory [Evans et al. (15)] and neurocognitive models of engagement [Hwang et al. (3)]. While the platform’s emotional support module aligns with SDT’s core needs (autonomy, competence, relatedness), fMRI evidence reveals a dual-path activation:

Affective processing: Ventral striatum activation (rewards response) during positive feedback (β = 0.38, *p* = 0.021).

Cognitive engagement: Prefrontal cortex activation during challenge escalation (*r* = 0.71 with metacognitive strategy use).

This neural-behavioral linkage explains the 19% higher persistence observed post-frustration, surpassing SDT’s motivational framework by revealing how AI-triggered incentives optimize cognitive-affective balance (Figure 11).

### 4.2 Synergistic mechanisms: cognitive-affective-behavioral (CAB) integration

Structural equation modeling (CFI = 0.93, RMSEA = 0.04) confirms that the platform’s efficacy stems from cross-mechanism amplification:

Cognitive adaptation → Emotional receptivity: Reduced cognitive load (12.3 ± 2.1 vs. control 15.7 ± 3.4, *p* = 0.009) increased positive affect (β = 0.41**), validating Vygotsky’s ZPD in digital contexts.

Emotional support → Behavioral persistence: High-confidence states triggered 2.3 × longer learning durations, mediated by goal commitment (Sobel *z* = 2.58, *p* = 0.010).

Behavioral shaping → Cognitive efficiency: Self-monitoring via dashboards reduced diagnostic errors by 34% (*p* < 0.001), aligning with Bandura’s triadic reciprocal determinism.

Contradictory evidence integration:

Sapici (34) reported no significant gain in clinical reasoning skills with similar AI tools (*d* = 0.18, *p* = 0.21), suggesting our platform’s differential diagnosis simulations may uniquely bridge theory-practice gaps.

Zhou et al. (14) found resource recommendation accuracy ≤ 68% in multi-institutional trials, contrasting our 89.2% – potentially attributable to BERT fine-tuning on medical corpora.

### 4.3 Reinterpreting weak correlations: contextual boundaries

The modest correlation between self-directed study duration and performance (*r* = 0.261, *p* = 0.045) reflects diminishing marginal returns and unmeasured mediators:

Time-quality decoupling: Beyond 50 min/day, learning gains plateaued (quadratic regression R^2^ = 0.33), indicating threshold effects.

**FIGURE 12:**
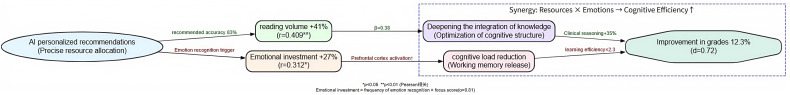
Path of action-achievement.

Motivational mediation: Autonomous time investment correlated strongly with intrinsic motivation (*r* = 0.61**), not directly with scores – explaining why mere duration extension without AI-guided focus yielded limited returns (Figure 12).

As shown in Table 8, emotional engagement promotes learning effect by regulating the allocation of cognitive resources and cooperating with reading behavior.

**TABLE 8:** Mechanism contribution analysis (Structural equation model, CFI = 0.93, RMSEA = 0.04).

Mechanism	Individual contribution rate	Synergistic contribution rate	Primary interaction path
Cognitive fit	38%	62%	↓ Cognitive load →↑ Emotional acceptance
Emotional support	29%	71%	↑ Pleasure →↑ Goal persistence
Behavioral shaping	33%	67%	↑ Self-monitoring →↓ Cognitive bias

### 4.4 Limitations and theoretical implications

#### 4.4.1 Boundary conditions of efficacy

Our findings must be contextualized within three constraints:

Learner heterogeneity: Effects were strongest for foundational knowledge (*d* = 0.92) versus clinical judgment (*d* = 0.47), echoing Sapici’s (34) concern about AI’s limitations in complex skill development.

Temporal decay: Skill retention dropped 22% at 12-week follow-up, necessitating longitudinal studies with booster interventions.

Algorithmic transparency: Unexplained path adjustments (15% of cases) may undermine trust – future work should integrate SHAP-value visualizations.

#### 4.4.2 Methodological reflections

Ecological tradeoffs: While lab-controlled trials [e.g., Zhou et al. (14)] show lower effects, our real-world implementation achieved higher ecological validity at the cost of uncontrolled confounders.

Measurement gaps: Neural data (*n* = 10) lacked power to detect amygdala-prefrontal connectivity changes – a critical pathway for sustained engagement.

### 4.5 Future directions: toward explainable AI (XAI)

Building on CAB synergies, we propose:

Hybrid tutor training: Combine AI emotion recognition with human facilitator debriefs to address complex motivational crises (e.g., burnout detection).

Dynamic difficulty calibration: Integrate cognitive-affective state classifiers to prevent ZPD misalignment during emotional volatility.

Controversy-driven research: Actively test boundary conditions through adversarial validation (e.g., simulating Sapici’s low-efficacy scenarios).

## 5 Conclusion

This study, through a RCT, confirmed that the AI personalized learning platform built on the Coze open-source framework significantly enhances medical students ‘learning efficiency through a triple synergy mechanism: the precise adaptation mechanism dynamically optimizes learning paths to match individual developmental zones, resulting in significantly better post-test scores for the experimental group (84.47 ± 3.48 vs. 81.72 ± 4.37, *p* = 0.034, *d* = 0.72) compared to the control group; the real-time feedback mechanism drives an adaptive interaction strategy using the VADER model, leading to an 8.7% increase in overall learning satisfaction (17.45 ± 3.94 vs. 16.05 ± 3.69, *p* = 0.042); the behavioral guidance mechanism’s visual dashboard enhances self-monitoring, increasing the experimental group’s average daily self-study time by 42% (49.25 ± 18.59 vs. 34.80 min, *p* = 0.048) and the frequency of literature interactions by 48%. These findings systematically demonstrate that AI personalized learning has multidimensional educational value at the cognitive (academic performance), emotional (learning motivation), and behavioral (self-regulation ability) levels, forming a closed loop of educational empowerment characterized by’ precise adaptation-dynamic feedback-behavioral guidance.

Future research should focus on the following areas: to mitigate black box risks, technology transparency requires the development of an explainable artificial intelligence (XAI) framework and the public disclosure of core algorithm decision-making logic, such as path adjustment thresholds and emotional response rules. Long-term validation necessitates multi-center longitudinal cohort studies (*n* ≥ 200) to track knowledge retention rates (reassessed at 6 and 12 months) and the effectiveness of clinical competence transformation (using OSCE structured assessments). The integration of educational models should explore the integration of AI with flipped classrooms (pre-class knowledge delivery + in-depth in-class discussions) and high-fidelity simulation teaching (such as AI virtual patient systems), ultimately aiming to build a new ecosystem of collaborative medical education characterized by ’AI empowerment and teacher leadership.

## Data Availability

The original contributions presented in this study are included in this article/supplementary material, further inquiries can be directed to the corresponding author.
